# Consensus statement on microglial and macrophage functions in gliomas

**DOI:** 10.1007/s00401-026-02999-3

**Published:** 2026-04-16

**Authors:** Yuqi Zheng, Islam Alzoubi, Manuel B. Graeber, Atul Anand, Lara Barazzuol, Mario Dorostkar, Karl Frontzek, Anna Golebiewska, Costas G. Hadjipanayis, Tibor Hortobagyi, Azzam Ismail, Gerard H. Jansen, Bozena Kaminska, Daniel Kirschenbaum, Yoshihiro Komohara, Bjarne Winther Kristensen, Kevin S. Lee, Emilie Le Rhun, Q. Richard Lu, Kaspar Matiasek, Christian Mawrin, Alessandro Michelucci, Nicola Montemurro, Marc-André Mouthon, Quan Liu, Regina Reimann, Roman Sankowski, Alejandro Schcolnik-Cabrera, Michael Schulz, Christian M. Schürch, Marius Schwabenland, Aurélie Tchoghandjian, Indrė Valiulytė-Simaitė, Mariano S. Viapiano, Tobias Weiss, Michael Weller, Max Wintermark

**Affiliations:** 1 Ken Parker Brain Tumour Research Laboratories, Brain and Mind Centre, University of Sydney, Camperdown, Australia; 2 School of Computer Science, The University of Sydney, Sydney, NSW 2008 Australia; 3 University of Sydney Association of Professors, University of Sydney, Sydney, NSW 2006 Australia; 4 Department of Clinical Medicine and Biotech Research and Innovation Center (BRIC), University of Copenhagen, Copenhagen, Denmark; 5 Department of Pathology, The Bartholin Institute, Rigshospitalet, Copenhagen University Hospital, Copenhagen, Denmark; 6 Department of Biomedical Sciences, University Medical Center Groningen, University of Groningen, 9700 AD Groningen, The Netherlands; 7 Department of Radiation Oncology, University Medical Center Groningen, University of Groningen, 9700 RB Groningen, The Netherlands; 8 Institut für Klinische Pathologie und Molekularpathologie der Region NÖ-Mitte (Universitätsklinikum St. Pölten), Dunant-Platz 1, 3100 St. Pölten, Austria; 9 Queen Square Brain Bank, UCL Queen Square Institute of Neurology, The National Hospital for Neurology and Neurosurgery, University College London Hospitals NHS Foundation Trust, Dubowitz Neuromuscular Centre, Great Ormond Street Hospital for Children NHS Foundation Trust, Queen Square, London, WC1N 3GB UK; 10 NORLUX Neuro-Oncology Laboratory, Department of Cancer Research, Luxembourg Institute of Health, 6A Rue Nicolas-Ernest Barblé, 1210 Luxembourg, Luxembourg; 11 UPMC Brain Tumor Program, Center for Image-Guided Neurosurgery, University of Pittsburgh Medical Center (UPMC), AANS/CNS Joint Section on Tumors, Rolling Meadows, USA; 12 Institute of Neuropathology, University Hospital Zurich, Zurich, Switzerland; 13 Histopathology Department, L5 Bexley Wing, SJUH, Leeds, LS9 7TF UK; 14 General Neuropathology, Creutzfeldt-Jakob Disease Consultancy and Neuropathology, Division of Diagnostic and Molecular Pathology, Laboratory Medicine Building Rm 123, The Ottawa Hospital - Civic Campus, Ottawa, ON K1Y 4E9 Canada; 15 Laboratory of Molecular Neurobiology, Nencki Institute of Experimental Biology, Pasteur 3, 02-093 Warsaw, Poland; 16 German Cancer Research Center (DKFZ), Im Neuenheimer Feld 280, 69120 Heidelberg, Germany; 17 Department of Cell Pathology, Graduate School of Medical Sciences, Kumamoto University, Kumamoto, Japan; 18 Department of Neuroscience, Department of Neurosurgery, and Center for Brain, Immunology, and Glia, University of Virginia, Charlottesville, VA USA; 19 Department of Medical Oncology and Hematology, University Hospital and University of Zurich, Rämistrasse 100, 8091 Zurich, Switzerland; 20 Department of Pediatrics, Brain Tumor Center, Brain Tumor Program EHCB, Cancer & Blood Diseases Institute Cincinnati Children’s Hospital Medical Center, Cincinnati, OH 45229 USA; 21 Section of Clinical & Comparative Neuropathology, Centre for Clinical Veterinary Medicine, Ludwig-Maximilians-Universitaet, 80539 Munich, Germany; 22 Institut für Neuropathologie, Otto-von-Guericke-Universität Magdeburg, 39120 Magdeburg, Germany; 23 Neuro-Immunology Group, Department of Cancer Research, Luxembourg Institute of Health, 6A, Rue Nicolas-Ernest Barblé, 1210 Luxembourg, Luxembourg; 24 Unit of Neurosurgery, Pisana University Hospital, Pisa, Italy; 25 Stabilité Génétique Cellules Souches et Radiations UMRE008, Université Paris Cité Université Paris Saclay, U1274, INSERM LRP/SDRR/iRCM/JACOB/DRF, CEA18, route du Panorama, BP n°6, 92265 Fontenay-aux-Roses Cedex, France; 26 Department of Neurosurgery, The Fourth Affiliated Hospital of Guangxi Medical University, Liuzhou, China; 27 Institute of Neuropathology, Medical Center, University of Freiburg, Freiburg, Germany; 28 Department of Medical Microbiology and Immunology, Faculty of Medicine and Dentistry, University of Alberta, Edmonton, AB Canada; 29 Department of Pathology and Neuropathology, University Hospital and Comprehensive Cancer Center Tübingen, Tübingen, Germany; 30 Aix-Marseille Univ, CNRS, INP, Inst Neurophysiopathol, GlioME Team, 27 Boulevard Jean Moulin, 13005 Marseille, France; 31 Laboratory of Molecular Neurooncology, Lithuanian University of Health Sciences, Neuroscience Institute, Eiveniu str. 4, 50161 Kaunas, Lithuania; 32 Department of Neuroscience & Physiology, SUNY Upstate Medical University, Syracuse, NY 13210 USA; 33 Department of Neurology, University Hospital and University of Zurich, Frauenklinikstrasse 26, 8091 Zurich, Switzerland; 34 Department of Neuroradiology, The University of Texas MD Anderson Cancer Center, Houston, TX USA; 35 Cluster of Excellence iFIT (EXC 2180) “Image-Guided and Functionally Instructed Tumor Therapies”, University of Tübingen, Tübingen, Germany

**Keywords:** Glioma, Microglia, Macrophages, Immunotherapy, Neurooncology

## Abstract

This international consensus statement synthesizes key findings on the complex roles of microglia and macrophages (tumor-associated microglia/macrophages or TAMs) in glioma progression and therapeutic resistance. Recent advances have highlighted the cellular, spatial, and temporal heterogeneity of TAMs, their functional plasticity, and the intricate interactions between TAMs, glioma stem cells, and the neuronal microenvironment, challenging the M1/M2 classification paradigm for TAMs in gliomas and other misconceptions. The statement emphasizes that glioma cells manipulate TAMs to suppress anti-tumor functions, while microglia-mediated modulation of neuron-glioma cell interactions promotes tumor progression. Furthermore, glioblastoma-derived extracellular vesicles (EVs) reprogram microglia to support tumor progression, offering novel therapeutic targets. To advance research and develop more effective treatments, the statement advocates for precision therapies targeting specific TAM subsets or functions, the use of bioengineered EVs as a therapeutic approach, and a shift away from simplistic terminology like “M1/M2” and “neuroinflammation”. Ultimately, this new understanding can support innovative strategies to modulate the tumor microenvironment, turning immunosuppression into immunostimulation and improving outcomes for patients with glioblastoma and other types of gliomas.

## Introduction to tumor-associated microglia/macrophages (TAMs) in gliomas

TAMs constitute a very significant component of the cellular microenvironment of gliomas and of glioblastoma in particular, which has the highest TAM:lymphocyte ratio among all cancers [68]. Following a period of relative neglect by neuro-oncologists, TAMs have become recognised as crucial players in the development and progression of gliomas in recent years [101, 104, 138].

It seems appropriate to differentiate between resident (pre-existing) microglia (MG) and newly arrived monocyte-derived macrophages (MDMs) in the tumor, despite their overlapping functions, as they have distinct developmental origins, markers, and potential roles in glioma development [127]. As will be discussed in detail, TAMs produce growth factors and cytokines, including chemokines, that promote tumor growth, survival, migration, and blood vessel formation, thereby creating a supportive environment for glioma cells to infiltrate surrounding tissue. Their interactions with glioma stem cells (GSCs) are considered to increase tumor aggressiveness and resistance to treatment [159].

As will also be discussed, targeting or modulating the functions of TAMs has been suggested as a promising new approach to improve treatment outcomes for patients with glioma [44, 165].

### There is cellular heterogeneity of TAMs in gliomas

*Section summary: TAMs in gliomas exhibit pronounced phenotypic and functional heterogeneity, originating from two primary sources: resident microglia and monocyte-derived macrophages. These distinct subsets are characterized by unique gene expression profiles and surface marker signatures. Furthermore, the tumor microenvironment (TME) is also inhabited by other immune cell types, including neutrophils and dendritic cells, which contribute to its complexity and diversity.*

The dual origin of TAMs in glioblastoma, deriving from either MG or MDMs, has been well studied [17, 85]**.** Single-cell RNA sequencing (scRNA-seq) analyses have demonstrated that *CCR2*, *CD45RA*, *CD141*, *ICAM*, *CD11B*, *TGFBI*, *FXYD5*, *FCGR2B*, *CLEC12A*, *CLEC10A*, *CD207*, *ITGA4*, and *CD209* are enriched in MDMs, whereas *CX3CR1*, *SALL1*, *HEXB*, *P2RY12*, and *TMEM119* are highly expressed in MG [4, 39, 85, 88, 103]. Of note, discrimination of cell ontogeny based on a set of markers alone is challenging in the face of evidence that MDMs tend to adopt MG-like phenotypes once exposed to the brain micromilieu [14, 26, 127]. The determination of TAM ontogeny requires orthogonal techniques in humans, including somatic variant- or chimerism-based fate mapping [84, 121]. Mass cytometry studies showed that the highly expressed TAM markers are those commonly present in most phagocytes (CD11c, CD64, HLA-DR, and CX3CR1) [120]. CNS-resident MG, infiltrating MDMs (CD64^+^, CD11c^+^, and CD11b^+^), and neutrophils (CD66b^+^ and CD16^+^) comprise up to 80% (± 18%) of leukocytes in gliomas. *P2RY12* was found to be expressed in MG, while *ITGA4* was expressed mostly in monocytes and MDMs [17]. *CD45RA, CD141*, *ICAM*, and *CD45RA* were differentially expressed by MDMs compared to MG.

Among MDMs, four subpopulations have been described, one of them with high levels of CD163, CD206, CD169 that likely represent phagocytic macrophages [39]. These markers are also found in CNS border-associated macrophages (BAMs). ScRNA-seq studies showed overexpression of *NAV3, SLC1A3, SIGLEC8* and *P2YRY12* in MG, and *TGFBI, ITGA4, FPR3, IFITM3* and *S100A11* in MDMs [85]. Another scRNA-seq study distinguished two transcriptionally distinct MG subsets, monocytes, MDMs, and dendritic cells (DCs) and demonstrated differential expression in MG from the tumor center and periphery. The authors found downregulation of inflammation-related genes in the peripheral MG (*FCGBP and CCL20*), downregulation of genes associated with canonical interferon (IFN) responses (*IFI6, IFI27, STAT1, ISG15*), cell proliferation (*STMN1*), and downregulation of *CD163* (a scavenger receptor) [103]. Recently, a population of TAMs expressing ANXA1 and HMOX1, driven by FOSL2, has been associated with high-grade glioma transformation [161]. Sub-populations of *XCR1*^+^*CLEC9A*^+^*CADM1*^+^ type 1 conventional dendritic cell (cDC1) and *FCER1A*^+^*CLEC10A*^+^*CD1C*^+^ type 2 conventional (cDC2), along with the *CCR7*^+^*LAMP3*^+^*SAMSN1*^+^ DCs exhibiting a gene signature reminiscent of migratory DCs (MigDCs) were defined [103].

### TAMs in gliomas—does the cell of origin matter?

*Section summary: In malignant gliomas, the spatial distribution of TAMs is dynamically influenced by the TME, with specific niches such as hypoxic and necrotic regions shaping their localization. Despite this heterogeneity, the functional roles of distinct TAM subsets remain poorly defined, which poses a significant challenge for developing effective therapeutic strategies that target immune modulation in glioblastoma.*

The innate immune system of the central nervous system (CNS) is represented by parenchymal MG and CNS BAMs [30]. Under physiological conditions, these cells maintain brain homeostasis. In glioblastoma (WHO grade 4, isocitrate dehydrogenase [IDH] wild-type), MG and BAMs acquire TAM features. In his pioneering observations of 1925, Wilder Penfield noted: “Microglia in various stages of migratory and phagocytic activity can be seen everywhere throughout the tumor” [97]. However, upon the onset of necrosis, the majority of TAMs in hypoxic tissue originate from MDMs rather than MG [68, 73]. Accordingly, MG appears to be more important in tumor areas where diffuse tissue infiltration is taking place [25, 166]. Thus, brain-derived TAMs are complemented and in some tumor areas replaced by myeloid cells from the circulation, which include monocytes, myeloid-derived suppressor cells (MDSCs), neutrophils, and DCs.

It has been proposed that these peripheral immune cells home in on the tumor in response to wound-like properties of the malignant tumor mass [33], including the upregulation of immune-mobilizing chemokines [34].

Collectively, TAMs account for more than 30% of all cells within the tumor mass of glioblastoma [82] and 80% of immune cells within the TME [39], creating transcriptionally and spatially diverse subsets [91, 103, 157]. The ratio between TAMs derived from MG and monocytes varies across patient tumors and in preclinical models, where distinct transcriptomic genetic and histopathological features of TAMs and their distribution largely depend on the spatial organization of tumor niches, which in glioblastoma is mainly driven by hypoxia [49]. Monocytic TAMs accumulate in hypoxic and necrotic areas with increased blood–brain barrier (BBB) leakage, and microglial TAMs are abundant at the tumor border and within invasive niches, respectively [103]. Compared to IDH wild-type glioblastoma, IDH mutant low-grade gliomas do not show necrotic, contrast-enhancing areas, with TAM origin consequently shifted towards MG [39, 59]. Interestingly, while circumscribed tumors create a visible accumulation of microglial TAMs at the tumor margin, more diffuse tumors display various activation states, including homeostatic and highly ramified MG, that coexist within the infiltrative tumor niche [157]. An increased density of microglial TAMs inside tumors, and not at tumor borders, positively correlates with survival [162]. It remains to be clarified if the pro-inflammatory features of microglial TAMs result simply from earlier stages of polarization against brain injury or whether they play an alternative active role in the maintenance of tumor growth. The exact origin of blood-derived TAMs is still unsettled since myeloid cells lose their identity markers (e.g., Ly6C). The infiltration of MDSCs into the tumor suggests the generation of new immature bone marrow (BM)-derived myeloid cells in response to tumor growth. Moreover, TAMs share somatic mutations with peripheral blood monocytes [161]. Undifferentiated monocytes show the strongest signals in hypoxic niches with a leaky BBB, toward heterogeneous monocytic TAMs in the tumor core [157], suggesting that these areas constitute their main entry niche. While the majority represent classical and non-classical monocytes, the infiltration of circulating MDSCs has also been reported [5, 45, 110]. Similarly, microglial TAMs downregulate homeostatic markers (e.g. *Tmem119*, *P2ry12*). Both entities can also be replenished by active proliferation within glioblastoma, as indicated by the presence of actively cycling TAMs. In mouse models of glioblastoma, adoptive transfer experiments suggest that the great majority of MDMs are derived from classical monocytes [103].

The advances in single-cell transcriptomics have enabled the investigation of TAM origins in greater detail, based on larger gene signatures, thereby reducing the bias associated with limited marker-based flow cytometry approaches [16, 57, 126]. Trajectory analyses in patient tumors, syngeneic mouse glioblastoma models, and patient-derived xenografts confirmed the active conversion of the majority of MG and monocytes toward highly immunosuppressive TAMs [91, 101, 103, 157]. Importantly, microglial and monocytic TAMs both contribute to and partly compensate for each other's functions [103], highlighting a potential challenge for TAM-targeting therapies. Additional compensatory mechanisms can be maintained by neutrophils, as shown upon depleting monocyte chemoattractants in *PDGFB*-driven genetically engineered mouse models of glioblastoma [23]. While recently identified immunomodulatory programs, including inflammatory and immunosuppressive states, are not equally distributed across TAMs of different cellular origins [84], it remains to be determined to what extent diverse TAMs can compensate for the spectrum of these states and functions. Whether therapeutic strategies should target the immunomodulatory functions of TAMs as a whole or in a manner specific to their cell of origin remains to be determined [100].

## Cellular composition and ontogeny

### Microglia and monocyte-derived macrophages have a dual role in gliomas: tumor-supportive and anti-tumor functions

*Section summary: MG and MDMs play dichotomous roles in glioma development, influencing both tumor growth and anti-tumor immune responses. Their functions are modulated by interactions with the TME, which can drive them towards immunosuppressive phenotypes that facilitate disease progression. Recent breakthroughs in spatial and multiomics technologies have enhanced our understanding of the polarization mechanisms governing MG and MDM behavior in gliomas, as well as their prognostic significance.*

MG and MDMs play a complex role in gliomas, exhibiting both tumor-supportive and anti-tumor functions. This dual nature is influenced by the tumor cells and/or the TME, which can convert TAMs into immunosuppressors that promote tumor growth and invasion. In response to the glioma microenvironment, MG and MDMs can release factors like Vascular Endothelial Growth Factor (VEGF) and express VEGF receptor 1 (VEGFR-1) [69] as well as IL-10, which support tumor progression. They are also recruited by chemoattractants, such as SDF1/CXCL12, MCP-1 (CCL2) [102] and CSF-1, that not only attract these cells but also promote their polarization (see Textbox 1) into a state that supports tumor growth [67]. To advance treatment results, further research into the polarization mechanisms and interactions between MG, MDMs, and the TME is necessary. The introduction of spatial and multiomics technologies into glioma research has led to important discoveries, such as the acquisition of a mesenchymal program in TAMs induced by their interaction with glioma cells [53], the enrichment of tissue maintaining (previously referred to as M2-like) macrophages (see Textbox 1) in perivascular niches in patients with glioblastoma with poor survival [153], and a multi-layered, structured TME where TAMs are enriched in the hypoxia-adjacent and angiogenesis niches [49], supporting the role of hypoxia as an important microenvironmental driver of immunomodulatory expression programs [9, 49, 84, 109]. These immunomodulatory programs, and their associated myeloid cell phenotypes, can predict response to immunotherapy and overall survival [84].

Functionally, the divergent roles of macrophages can be understood by considering their dual responsibilities as both guardians of tissue homeostasis and immune defenders. Therefore, MG also possesses intrinsic anti-tumor properties, particularly in the early, but also at later stages of glioma development [84]. When activated and in a pro-inflammatory state, they can promote immunity against glioma cells. The exact timing, signals, and spatial cues that switch MG and macrophages from anti-tumor to tumor-supportive roles remain unclear. Tumor-associated factors such as hypoxia-associated nutrient deprivation are likely culprits [49, 153]. Understanding this transition seems important, especially for developing immuno-therapeutic strategies.

### Hypothesis of gliomas as wounds that do not heal

*Section summary: Gliomas, especially those harboring IDH mutations, display characteristics reminiscent of a chronic, non-healing wound, marked by persistent remodeling of the extracellular matrix (ECM) and profound metabolic dysregulation. This aberrant microenvironment is characterized by the accumulation of oncometabolites, such as 2-hydroxyglutarate (2-HG), which in turn drive tumor invasiveness, angiogenesis, and immune evasion. Furthermore, these ECM alterations also contribute to the development of treatment resistance, with distinct ECM subtypes correlating with divergent clinical outcomes, underscoring the complex interplay between the TME and disease progression.*

Gliomas, particularly IDH-mutant variants, are increasingly recognized as tumors driven by mechanisms resembling a wound that does not heal, largely due to chronic ECM remodeling and the accumulation of the oncometabolite 2-hydroxyglutarate (2-HG). This “wound-like” behavior is marked by persistent alterations in the ECM, where continuous deposition of components such as hyaluronic acid, tenascin C, and collagen promotes tumor invasiveness, angiogenesis, and immune suppression [149]. For instance, tenascin C expression is linked to reduced mTOR signaling in infiltrating immune cells, which restricts their proliferation, a mechanism that can undermine the immune response within the TME [149].

IDH mutations, prevalent in approximately 10% of gliomas [96], particularly in hypoxic and acidic TME from grade 3 anaplastic astrocytomas and oligodendrogliomas, result in the accumulation of 2-HG, which disrupts cellular processes. Elevated 2-HG levels inhibit α-ketoglutarate–dependent dioxygenases, leading to widespread epigenetic changes, including DNA and histone hypermethylation [112]. These changes enhance malignant proliferation and promote features such as mesenchymal transition, further contributing to tumor progression. Moreover, 2-HG stabilizes hypoxia-inducible factor (HIF)-1α at the protein level, driving angiogenesis and impairing immune cell recruitment by reducing CXCL10 expression [40, 112]. This combination of metabolic reprogramming and ECM alterations forms a unique, self-sustaining, and constantly evolving environment that supports tumor growth and evasion of immune surveillance.

The ECM remodeling in gliomas is not only driven by tumor cells but also by stromal components, which increase ECM stiffness and exacerbate treatment resistance [149]. This pro-stiffness environment, compounded by the secretion of collagen VI, creates barriers to effective therapies, such as bevacizumab, and contributes to a poor prognosis [31]. Additionally, ECM changes actively modulate the immune landscape, with hyaluronan binding to receptors like CD44 and CD168, activating pathways that promote tumor invasion and immune tolerance through macrophage re-education via elevated expression of PD-L1 [31].

Recent studies led by Wei et al. [151] have further refined our understanding of ECM-related subtypes in IDH-mutant gliomas. Two major subtypes, ECM1 and ECM2, have been identified based on distinct ECM signatures. While the ECM2 subtype was reported to present a more favorable prognosis with higher mutation profiles in *FUBP1*, ECM1 subtypes, enriched with mutations in *TP53, CDKN2A/B, NF1*, and *EGFR*, were associated with poor prognosis due to enhanced ECM remodeling, EMT (epithelial–mesenchymal transition), and increased immune infiltration, including resting DCs and macrophages [151]. Additionally, ECM1 was linked to dysregulated metabolic pathways, such as amino acid and lipid metabolism, which further promote tumor survival and growth [151].

## Context-dependent functional states of microglia and macrophages in glioma

### The spatial distribution of microglia and macrophages in gliomas plays a role in their functional behavior

*Section summary: The spatial topography of MG and MDMs within gliomas has a profound impact on their functional repertoire, with regional specialization emerging in response to tumor-intrinsic factors such as hypoxia, architectural patterning, and microenvironmental heterogeneity. Therapeutic interventions can reconfigure these spatial distributions, underscoring the interplay between the TME, cellular subsets, and treatment modalities that collectively shape glioma progression and outcome.*

The spatial distribution of MG and MDMs within tumors appears to play a significant role in their functional behavior. MG of the ramified phenotype are typically found at the tumor margins (diffuse infiltration zone), where they may facilitate invasion [56] and gradually switch to pro-tumorigenic ameboid TAMs. TAMs derived from MDMs are especially numerous in both necrotic zones in the center of a malignant glioma and in perivascular areas, close to the compromised BBB, controlling chemokine dynamics [19, 27, 165]. MDMs in hypoxic tissue areas are reprogrammed for immunosuppression [122]. It is interesting to note, however, that TAMs present in the dense tumor areas arise both from MG and MDMs [79, 84]. Importantly, the ratio between MG- and monocyte-derived TAMs can vary depending not only on the tumor niche and stage of tumor development but also on inter-patient differences. Such diversity is preserved in in vivo preclinical models with different genetic and histopathological features, where syngeneic models create bulky tumors with a high proportion of monocytic TAMs, whereas patient-derived xenografts sustain TAMs largely from MG, and genetically engineered mouse models show varying ratios depending on the genetic background [19, 91, 103, 157]. Recent studies utilizing state-of-the-art omics approaches impressively demonstrated that organization of glioblastoma architecture follows either high or low structuring, which seems to be driven by tumor individual cues [49, 115]. Not surprisingly, glioblastoma samples with a higher structured architecture possessed more and larger vessels. Consequently, the distribution of TAMs in glioblastoma varies from patient to patient, but follows basic rules of tumor-intrinsic spatial regionalization: infiltrating tumor area, solid/tumor-rich region, vascularized tumor, and hypoxic zones. A recent study [52] utilized imaging mass cytometry to determine different myeloid states across glioblastoma regions and reports multiple MG and MDM subsets. Furthermore, they describe that one of the main drivers of myeloid spatial organization was hypoxia, and that this compartimentalization is a conserved pattern across patient samples [52]. Treatments can also modify the spatial organization of MG within tumors. For example, after tumor surgery, the transient increase in BBB permeability is associated with an increase in MG in the core region of the tumor upon recurrence [12]. Further, corticosteroids skew MG phenotypes [84], but the exact effect on polarization is context- and corticoid receptor-dependent. It is also important to distinguish between long-term changes at recurrence in patients [4], where a subset of patients shows an increase in TAMs and the acute reaction to treatment (including resection, glucocorticoids, as well as radiotherapy and chemotherapy). Interestingly, while previous efforts attempting to tackle tumor-promoting features of TAMs resulted in enhanced survival in preclinical models, multi-modal treatment strategies, including TAM depletion by BLZ945, have been recently shown to induce the formation of a tumor stem cell niche by fibrotic responses. Yet, those niches were devoid of CD68^+^ TAMs [147]. These findings imply that TAMs not only promote tumor growth through multiple mechanisms, but also that their deficiency can be compensated for by other cellular processes, leading to tumor recurrence. In summary, it appears that tumor architecture is primarily determined by intrinsic traits of the tumor itself, while the interplay between infiltrating tumor-stroma cells, including TAMs, and the TME gives rise to distinct micro-niches.

### Microglia functions converge towards those of classical activated macrophages in gliomas

**Fig. 1:**
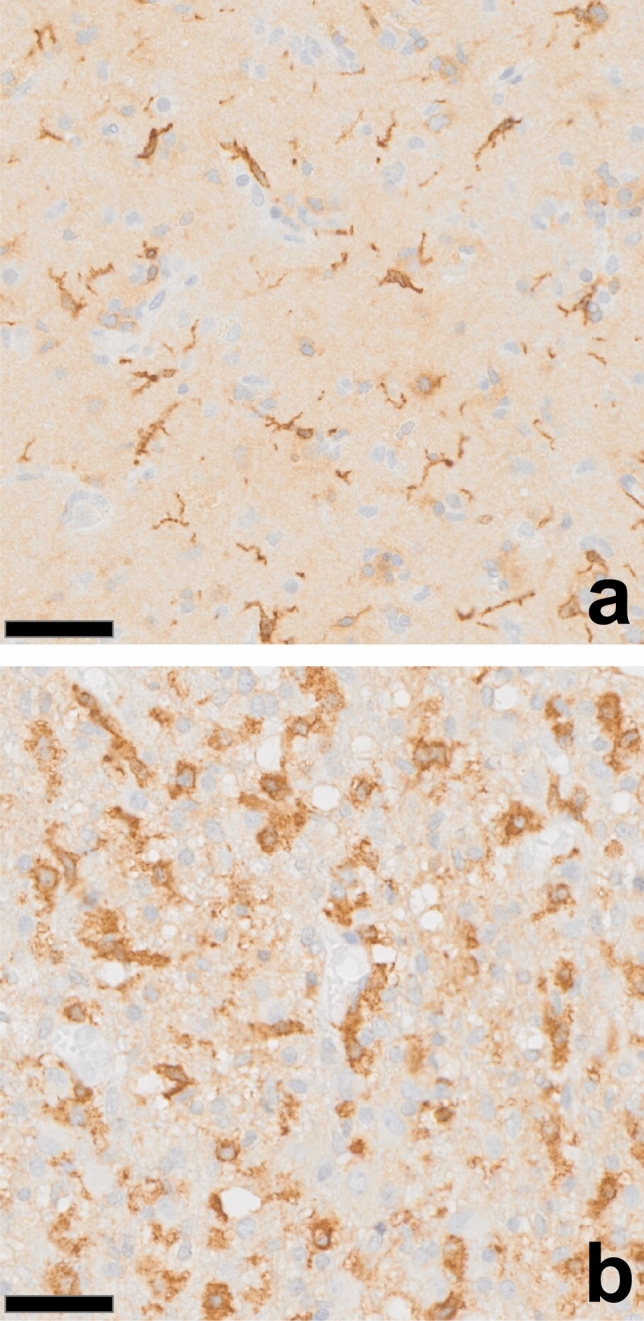
CD163 labeling of MG in glioblastoma. **a** Frontal cortex in a glioblastoma case showing de novo upregulation of CD163 in ramified microglia, a phenomenon rarely seen to this extent in other human diseases. **b** Glioblastoma-infiltrated brain tissue displays presumed MG with macrophage characteristics: stout, ramified processes and f*airly regular intercellular spacing similar to normal brain tissue* although cell density is increased. Note the absence of significant macrophage clustering. Brown, diaminobenzidine reaction product. Scale bar: 50 microns. Courtesy Y. Zheng/M.B. Graeber

*Section summary: In the context of gliomas, activated microglia undergo a significant functional shift, adopting characteristics reminiscent of classically activated macrophages. This phenotypic transformation is driven by pro-inflammatory cytokines, resulting in enhanced phagocytic activity and antigen presentation capabilities. However, microglia can also express CD163 in glioblastoma, a marker normally found on monocyte-derived macrophages* (Fig. 1)*.*

Under homeostatic conditions, MG represent a unique immune cell population distinct from other tissue-resident macrophages [135]. This specific identity stems from their developmental origins, maintenance, unique gene expression signature, and environmental specialization [14, 26]. MG originate from embryonic yolk sac progenitors that migrate to the brain during development and self-renew postnatally independently of peripheral blood cells [63, 140]. In contrast, most tissue macrophages derive from fetal hematopoietic stem cells and are replenished by blood-derived monocytes. During development, MG display an amoeboid morphology and are essential for neuronal development and synaptic pruning [94], whereas they acquire a ramified state after birth, characterized by a small soma and long process extensions possessing highly branched ramifications that scan the brain parenchyma. MG signatures are mainly influenced by CNS factors, including TGF-β and IL-34, which enable the acquisition of a specific homeostatic signature (also defined as the sensome signature) characterized by unique lineage markers, including TMEM119, SALL1, P2RY12, and epigenetic landscapes tightly linked to neuronal activity [11, 45, 47]. Similarly, tissue macrophages outside the CNS develop unique programs tailored to local stimuli, such as microbiota in the gut or heme metabolism in the liver. Notably, the MG homeostatic signature is reduced in their activated states, such as during infections and in gliomas [136, 157], showing a certain degree of functional convergence towards cytoprotective macrophages. However, many, if not most, macrophages in necrotic glioma tissue areas derive from bone marrow hematopoietic stem cells via circulating monocytes that infiltrate the tumor and differentiate into MDMs. They have larger somata but are smaller in terms of process reach, less branched, and they are migratory cells. MDMs normally express CD163, but MG are also capable of upregulating CD163, especially in gliomas of higher grades (Fig. 1), and they are able to transform into fully developed phagocytes. At this stage, they are morphologically indistinguishable from MDMs that have invaded the tissue. MG are CD49d (encoded by *Itga4*) negative, which can be used for identification by flow cytometry [91].

### A distinct activated state of TAM polarization is present in glioblastoma

*Section summary: Glioblastoma TAMs demonstrate a unique polarization state influenced by the TME, diverging from conventional M1 and M2 classifications.*

Textbox 1 summarizes information on the canonical M1 and M2 states and highlights that TAMs do not fit neatly into these categories. The following section discusses evidence suggesting that TAMs exist in a distinct, tumor-specific polarized state that does not align with the conventional reparative or pro-inflammatory programs of myeloid cells, and instead, appears to be shaped by the unique characteristics of the TME. A more detailed understanding of TAM biology, acknowledging the complexities of their role in cancer progression, is needed. Specifically, recent evidence suggests that TAMs in glioblastoma exist in a spectrum of activated states that is induced by the TME. This concept is supported by several studies, including a pan-cancer analysis [13, 150]. Earlier omics-based research also revealed that TAMs do not fit into the traditional and simplistic M1 or M2 categories, hinting at a more complex polarization state [137]. More recently, TAMs have been assigned to a distinct “mesenchymal macrophage” category [53]. This MES TAM population was characterized by an upregulation of genes reminiscent of mesenchymal glioma states, including *Vim, CD44, and ANXA1.* The authors proposed a reciprocal upregulation of corresponding markers in glioma cells but also TAMs, highlighting the co-evolution of GBM and its TME. Furthermore, co-culture experiments with glioblastoma cells have successfully induced tumor-specific polarization of macrophages, providing direct evidence for the tumor’s ability to shape the TAM phenotype [7, 54]. These findings collectively support the idea that glioblastoma TAMs occupy a novel functional state that is shaped by their interaction with the tumor.

### Microglia and macrophages in glioma-affected brains exhibit a spectrum of functional states

*Section summary: In glioma-affected brains, microglia and macrophages exhibit a spectrum of functional states, primarily adopting an anti-inflammatory, tumor-supportive phenotype that fosters immunosuppression, angiogenesis, and tumor progression. Importantly, they retain the ability to shift their polarization in response to environmental cues, impacting therapeutic responses and contributing to recurrence.*

MG and macrophages in a brain affected by glioma outside the local TME, i.e., not yet fully under the glioma’s regulation, may also exist in a spectrum of functional states, ranging from pro-inflammatory and anti-tumor (M1-like) to anti-inflammatory and tumor-supportive (M2-like) [44, 119]. The former (M1-like) is characterized by the production of cytokines like TNF-α, IL-6, and CXCL10, and the expression of high levels of MHC class II molecules for antigen presentation. However, if a true M1-like stage occurs in vivo in the case of glioma, this response is not associated with successful tumor suppression. In contrast, the other extreme state, M2 polarization, can be induced by cytokines in vitro, including IL-4, IL-10, CSF-1 and IL-13, and is characterized by the production of anti-inflammatory cytokines (e.g., IL-10, TGF-β) and growth factors like EGF (epidermal growth factor). This state promotes immunosuppression, tissue remodeling, angiogenesis, and tumor invasiveness [165]. It is typically characterized by the expression of markers, such as CD163, CD204, and CD206.

It seems safe to say that glioma-associated MG and macrophages predominantly adopt states closer to the conventional M2 phenotype, which is promoted by glioma cells. This is considered to create an immunosuppressive TME that fosters glioma growth and immune evasion. However, both types of TAMs appear to have maintained their ability to transition between polarization states in response to environmental cues. The M1-to-M2 shift facilitates glioma progression by inhibiting cytotoxic T cell activity, enhancing angiogenesis, and promoting tumor invasion. Thus, a vicious cycle is created where glioma survival and expansion are reinforced. Importantly, changes in TAM polarization following treatments influence therapeutic responses and contribute to recurrence. Cancer treatments themselves can lead to innate immune memory, as demonstrated by radiotherapy leading to MG priming [145]. Therapeutic strategies to manipulate TAM polarization need to take these effects into account. Landmark studies utilizing preclinical glioma models demonstrated that targeting TAMs via CSF1-CSF1R axis inhibition resulted in depletion of a majority of macrophages, resulting in improved survival. However, the remaining cells became re-polarized, ultimately leading to tumor recurrence. Interestingly, overcoming these resistance mechanisms was achieved by a combination of radiotherapy [4, 104, 106].

### Same, Same but different? Lessons from brain metastasis-associated macrophage populations

*Section summary: Despite sharing the same host organ, brain metastases (BrM) and gliomas exhibit distinct TMEs that are primarily shaped by intrinsic properties of the tumor itself, rather than the brain context. Notably, TAMs in BrM, regardless of their origin from MDMs or resident MG, play a key role in establishing immunosuppressive and pro-tumoral TMEs. The transcriptomic profiles of these TAMs are linked to tumor-specific features, underscoring the importance of tumor origin in defining the immune landscape of brain metastases. These findings have implications for the development of therapeutic strategies, suggesting that targeted interventions against TAMs may need to be tailored to specific tumor types.*

IDH–wildtype glioblastoma is the most common primary tumor arising within the brain parenchyma; however, brain metastases (BrM) constitute the most prevalent brain tumor entity in adults. Up to 40% of all cancer patients are estimated to develop BrM over the course of their disease. Although virtually any malignancy can seed the brain, BrM most frequently originates from lung, breast, and cutaneous melanoma primaries [2, 105, 124]. Despite sharing the same host organ as gliomas, BrM co-evolve a strikingly distinct TME. As in gliomas, the BrM TME is dominated by myeloid cells [39, 59, 123]. Molecular differences among TAMs in BrM have been described in mouse models [60, 90, 125] and in human specimens [39, 59]. The identification of lineage markers, including CD49d encoded by *ITGA4/Itga4*, has enabled the dissection of macrophage ontogeny across BrM from diverse primary tumor entities. Notably, transcriptomic TAM programs in these studies have been linked to pro-tumoral functions in both monocytic and microglial TAMs. For example, Klemm et al. [60] showed that pharmacologic CSF1R blockade with BLZ945 reduced the number of extravasating tumor cells in two breast cancer BrM mouse models and transiently impeded BrM progression. Consistent with this, Rodriguez-Baena et al. [114] reported that genetic depletion of MG decreased BrM burden in melanoma models. They further demonstrated that targeting NF-κB signaling in microglial TAMs reprogrammed tumor-educated MG towards a more pro-inflammatory state, thereby enhancing antitumor, T cell-mediated immunity. Seminal cross-tumor analyses by Friebel et al. [39] and Klemm et al. [59] underscored that tumor-intrinsic properties, rather than the brain tissue context per se, primarily shape the immune landscape. By integrating transcriptomic and proteomic TAM datasets, both groups found entity-specific myeloid states. Using surface-marker-guided trajectory analyses, Friebel et al. [39] showed that IDH-wildtype gliomas harbor increased MDM populations triple-positive for CD163, CX3CR1, and CADM1, whereas distinct MDM subsets predominate in carcinoma- and melanoma-derived BrM. In contrast, IDH-mutant (lower grade) gliomas possess a higher number of microglial TAMs. Melanoma BrM exhibits the highest frequencies of infiltrating lymphocytes among analyzed tumor types and concurrently contains MDM subsets with elevated PD-L1 and PD-L2 expression, aligning with the greater abundance of regulatory and exhausted T cells within the TME. A recent pan-BrM scRNA-seq study by Xing et al. [155] corroborated these observations, reporting the highest levels of exhausted T cells in melanoma BrM, characterized by increased expression of canonical markers including *PDCD1*, *CTLA4*, *LAG3*, and *HAVCR2*. Extending beyond inter-tumor differences, Xing et al. further resolved intra-entity heterogeneity: patients with EGFR-mutant lung adenocarcinoma (LUAD) BrM displayed more pro-tumoral myeloid states than those with EGFR–wildtype LUAD. Consistent immunogenomic divergence in BrM TMEs has also been described in lung and breast cancer cohorts [8]. Collectively, these large-scale studies compellingly demonstrate that tumor-intrinsic features are the principal drivers sculpting the brain metastatic TME, surpassing the influence of the brain milieu itself. While the overall TME composition in various brain tumors of primary or secondary origin varies by the underlying tumor type, BrM harbor an enormously heterogenous pool of resident and monocyte-derived macrophages as well. Altogether, all TAM populations contribute to an immunosuppressive and pro-tumoral TME in BrM in a spatio-temporal manner.

## Dialogues within tumor niches favor tumor progression

### TAMs are key players in tumor angiogenesis and blood vessel remodeling in glioma

*Section summary: TAMs are key orchestrators of glioma progression, primarily through their ability to promote angiogenesis and remodel blood vessels within the TME. This is achieved *via* the secretion of pro-angiogenic factors, including vascular endothelial growth factor (VEGF) and chemokine (C-X-C motif) ligand 2 (CXCL2), as well as ECM remodeling. Notably, hypoxic conditions prevalent in gliomas further potentiate TAM activation, creating a self-reinforcing cycle that accelerates tumor growth and exacerbates tissue damage, ultimately driving malignant progression.*

MG and macrophages secrete pro-angiogenic factors such as VEGF and CXCL2, thereby facilitating and stimulating the growth of new blood vessels. The depletion of MG has been shown to significantly reduce tumor blood vessel density, highlighting their important role in this process. Moreover, MG dynamically interact with the tumor vasculature, particularly in perivascular regions, where they facilitate vessel growth and remodeling. Additionally, they upregulate matrix metalloproteinases (MMPs), such as membrane type 1 MMP and MMP-9, which can degrade the ECM and enhance angiogenesis and tumor invasion. However, rather than focusing solely on ECM degradation, it may be more accurate to consider the process of ECM remodeling by TAMs, which not only degrade pre-existing ECM components via MMPs, but also produce new ECM proteins that promote angiogenesis and glioblastoma progression. This concept is supported by studies on osteopontin/SPP1 in the glioblastoma ECM [20, 148]. While it has been suggested that glioblastoma macrophages may also deposit other ECM components, such as collagen and hyaluronan, direct evidence for this is limited compared to other tissues and cancers. Glioma-derived factors upregulate membrane type 1 MMP in microglia, which in turn activates MMP-2 produced by glioma cells to cleave ECM and facilitate tumor cell spread [81].

Insufficient blood supply within glioblastomas leads to hypoxic and necrotic tissue areas. The hypoxic environment perpetuates a vicious cycle, driving TAM recruitment and activation, which in turn accelerates tissue remodeling, damage, and tumor progression.

### Glioma cells manipulate TAMs to suppress anti-tumor functions and maintain GSCs

*Section summary: Glioma cells exploit TAMs to dampen anti-tumor responses and sustain GSC populations, which are essential for tumor progression and therapy resistance. This complex manipulation involves specific molecular mechanisms, including SPP1/POSTN-integrin interactions, hypoxia-induced signaling pathways, and alterations in metabolic processes.*

The TME significantly influences the behavior of TAMs. Glioma cells are not only capable of manipulating TAMs by inhibiting their anti-tumor functions but also suppress T cell activity and limit the recruitment of additional macrophages. Conversely, TAMs play a supportive role in glioma development by maintaining GSC populations. They achieve this by secreting factors that activate signaling pathways associated with stemness, thereby promoting glioma cell proliferation, survival, and therapy resistance [128]. Indeed, genetically engineered mouse models in which TAMs cannot provide trophic support to the tumor cells result in reduced glioma growth and improved animal survival [128]. Glioma-derived secreted phosphoprotein 1 (SPP1/osteopontin) and GSC-derived periostin (POSTN) through integrin αvβ3 on myeloid cells recruit and reprogram TAMs, as blocking this signaling by gene knockout or an RGD peptide inhibited TAM accumulation, tumor growth, and increased survival of mice bearing glioma [35] or human GSC xenografts [167]. A synthetic peptide 7aaRGD targeting SPP1-integrin interactions efficiently blocked microglia-dependent invasion of human and mouse glioma cells in vitro and prevented the emergence of immunosuppressive TAMs and led to normalization of peritumoral blood vessels when intratumorally delivered. Combining 7aaRGD with anti-PD-1 antibody resulted in reduced tumor growth, with an increase in the number of proliferating, interferon-ɣ producing CD8^+^T cells and concomitant depletion of regulatory T cells [36].

Hypoxia induces TAMs to support angiogenesis and glioma cell invasion via HIF-1α-mediated pathways and secretion of factors such as MIF and VEGF. Tumor-derived ligands such as versican can activate Toll-like receptors (e.g., TLR2) on MG, leading to the upregulation of enzymes that remodel the ECM and facilitate tumor spread [71]. Metabolic changes, including lactate accumulation [77], additionally polarize TAMs towards tumor-supporting states.

### Reciprocal molecular interactions between TAMs and GSCs sustain stemness, drive invasion, and foster therapeutic resistance in glioblastoma

*Section summary: The interactions between TAMs and GSCs are important for maintaining GSC stemness, promoting tumor invasion, and conferring therapeutic resistance. These interactions are mediated by various signaling pathways, including TGF-β1 and PTN. However, the relationship between TAMs and GSCs is complex and context-dependent, with evidence suggesting that TAMs can exert both promotive and inhibitory effects on GSC properties.*

TAMs and GSCs are found in perivascular and hypoxic regions as well as along invasive margins, where their proximity reflects a reciprocal molecular interaction critical for tumor progression. Despite the proven intimate interaction between GSCs and TAMs in the TME, contradictory results have been reported pertaining to the promotion and inhibition of GSCs features and glioma progression driven by TAMs interaction with GSCs.

TAMs are thought to support GSC maintenance and exert potent tumor-promoting effects within the microenvironment by sustaining stemness, promoting invasion, and facilitating resistance to therapy. Specifically, TAMs support the invasive capacity of GSCs via paracrine secretion of transforming growth factor beta 1 (TGF-β1) and activation of the TGF-β1–TGFBR2 signaling axis, in which MMP-9 acts as a pivotal downstream effector mediating enhanced invasiveness [159]. In addition, TAM-derived TGF-β1 binds to integrin αvβ5, increases phosphorylation of tyrosine kinase Src and subsequently the phosphorylation of Stat3. The activation of the αvβ5–Src–Stat3 pathway maintains GSC proliferation and stemness, while blockade of this axis attenuates the tumor-promoting functions of TAMs and extends survival in preclinical models [98]. Similarly, TAMs secrete pleiotrophin (PTN), a heparin-binding glycoprotein, which stimulates GSCs through its receptor PTPRZ1, thereby promoting GSC maintenance and tumor progression via the PTN–PTPRZ1 paracrine axis [128]. Furthermore, TAM-derived macrophage receptor with collagenous structure (MARCO) has been shown to reprogram non-mesenchymal GSCs into a more aggressive mesenchymal phenotype, characterized by enhanced invasion, migration, and resistance to ionizing radiation [116]. Together, these findings suggest that TAMs act as essential regulators of GSC function, operating through multiple signaling mechanisms to sustain stemness, drive invasion, and foster therapeutic resistance in glioblastoma.

On the other hand, however, co-culturing MG with patient-derived glioblastoma spheroids, which are enriched in GSCs, has been shown to block the invasive capacities of neoplastic cells [21]. A possible explanation for this diametrical phenomenon could lie in the microRNAs (miRNAs) expressed by GSCs during their interaction with MG and other TAMs. miRNA-504, which is highly expressed in neural stem cells and exhibits anti-tumor and anti-stemness properties both in vitro and in vivo, is naturally found to be downregulated in glioblastomas and GSCs. However, when overexpressed in GSCs, miRNA-504 diminishes the expression of stemness markers such as Nanog and Oct4, limits their self-renewal and neurosphere-forming capacities, and induces a switch of MG towards a pro-inflammatory phenotype, characterized by increased expression of CD86 and TNF-α [15].

The reciprocal interaction between TAMs and GSCs has recently been demonstrated [50]. The authors demonstrated that pro-inflammatory signals, mediated by TNF-α, induce the maintenance of GSCs and promote glioma progression by activating EGFR and AKT signaling pathways. This activation led to the production of TNFAIP6, which in turn can promote TAM immunosuppression. This inter-relationship has also been shown by the work of the Akkari team, revealing that a specific subset of TAMs can fuel tumor cells with lipid recycling [61].

### Microglia-mediated modulation of neuron-glioma cell interactions: a potential novel mechanism of tumor promotion

*Section summary: Microglia influence neuron-glioma interactions by modulating synaptic connections, potentially promoting tumor growth through brain-derived neurotrophic factor (BDNF)–tropomyosin receptor kinase B (TrkB) signaling. Additionally, microglia contribute to neuronal hyperexcitability in the glioma environment*,* highlighting the complex interplay between immune cells, neurons, and cancer cells.*

The interaction between glioma cells and neurons is a complex process. Neurons form synaptic contacts with glioma cells, and glutamatergic signaling promotes tumor proliferation and invasiveness [38]. Neuronal activity also drives tumor growth through paracrine signaling, e.g., via BDNF (brain-derived neurotrophic factor) and neuroligin-3 (NLG3) signaling [144]. Mechanisms of synaptic plasticity that are normally important for learning and memory in the healthy brain are controlled by glioma and enhance malignant growth. The BDNF-TrkB signaling pathway is central to this process, regulating both the strength and number of neuron-to-glioma synapses [139]. MG are considered to contribute to neuron-glioma cell interactions by influencing the formation of aberrant synapses between neurons and glioma cells [93, 107]. As part of their natural function, MG are involved in synapse formation and pruning, which can be exploited by gliomas. For instance, MG may facilitate the formation of glutamatergic synapses between neurons and glioma cells, allowing for the transmission of signals that promote tumor proliferation. By modulating synaptic connections, MG can indirectly influence the behavior of glioma cells and contribute to their progression. Furthermore, glioma-associated MG have been found to potentiate neuronal hyper-excitability in the glioma environment [160]. There is dysregulated microglial synaptic engulfment in diffuse midline glioma, potentially allowing modulation of tumor-associated neuronal hyperexcitability by targeting aberrant microglial synaptic engulfment [80].

## Extracellular vesicles (EVs) orchestrate glioma-brain cell interactions

### Microglia-derived EVs regulate neuronal activity and synaptic plasticity

*Section summary: Microglia-derived EVs play a role in regulating neuronal activity and synaptic plasticity, conveying molecular signals that maintain synaptic health under physiological conditions. In the context of glioma, EV-associated microRNAs, such as miR-21, are significantly upregulated and can be detected in peripheral biofluids, including plasma and urine. They are largely attributable to non-tumoral cells, particularly activated microglia*,* and may provide a novel avenue for early disease detection and monitoring.*

EVs, cell-derived lipid bilayer structures released into the extracellular space, play crucial roles in intercellular communication. EVs' roles have been minimized as mere waste disposal pathways that all cells have, but they are now recognized as highly complex mechanisms of cell-to-cell communication, antigen presentation, and signal transduction that can manipulate the fate of recipient cells in both health and disease. Indeed, EVs, which include exosomes, microvesicles, and apoptotic bodies, carry molecular cargo from source cells, such as proteins, RNA species, DNA, and lipids, all of which manipulate target cell behavior starting at the membrane interaction. Since an in-depth discussion of EVs is beyond the scope of this article, readers are invited to consult the following references [32, 65] for further information. While EVs can interact with cellular elements in the vicinity of source cells, they are also capable of traveling through circulation and bidirectionally crossing the BBB to interact with the brain parenchyma [77]. Since EVs mirror the status of source cells, it is not surprising that during healthy conditions, MG-derived EVs modulate synapse homeostasis and thus neuronal activity via specific cargo such as miRNA146a-5p, as well as neurotransmission promoting the neuronal synthesis of sphingosine and ceramides. Oligodendrocytes clear their excessive membranes by releasing EVs, which are then internalized by MG via macropinocytosis, without impacting their cytokine profiles [152]. When activated, MG release a greater number of EVs, which are also larger in size, suggesting a higher cargo capacity aimed at their target cells [158]. For example, in response to viral infections, MG release vesicles loaded with dsDNA fragments that affect IFN-α-responsive neurons, triggering apoptosis and thus neurodegeneration [10]. Upon damage, neurons secrete EVs loaded with miRNA-124, which when taken up by MG, stimulate a pro-inflammatory M1-like state (cf. Textbox 1) distinguished by elevated levels of MHC-II, IL-1β, nitric oxide, and TNF-α [99]. Beyond acute inflammatory signaling, microglia-derived EVs also play a role in tumor-immune communication. In glioma, EV-associated microRNAs such as miR‑21 are strongly upregulated and detectable in peripheral biofluids, including plasma and urine. Importantly, this signal originates largely from non-tumoral cells, particularly activated microglia, highlighting microglial EVs as early mediators of tumor-associated signaling and promising candidates for minimally invasive glioma biomarkers [29].

### Glioblastoma-derived EVs reprogram microglia to support tumor progression

*Section summary: Glioblastoma-derived EVs shape the TME by transporting mutant mRNAs, miRNAs, and immune checkpoint molecules. Upon uptake by microglia, these EVs reprogram their function, inducing M2-like polarization and suppressing anti-tumor immunity. This, in turn, fosters an immunosuppressive microenvironment that promotes tumor progression and enhances malignancy. Conversely, bioengineered EVs have emerged as a promising therapeutic strategy against glioblastoma.*

MG can be reprogrammed by glioblastoma-derived EVs to support tumor progression and limit survival. For instance, glioblastoma-derived EVs can transport tumor-specific mutant mRNAs and miRNAs related to biological processes such as angiogenesis, cell proliferation, histone modification, cell migration and immune responses [6, 129], as well as immune checkpoint molecules like PD-L1 and FasL, suppressing anti-tumor immune responses while promoting malignant survival and progression [87, 109, 113]. In gliomas, these vesicles can alter MG function, promoting immunosuppressive niches. Specifically, glioblastoma-derived EVs recruit MG to the TME, reducing their expression of thrombospondin-1, a negative regulator of angiogenesis [141]. Moreover, while glioblastoma-derived EVs loaded with hsa-miRNA-27a-3p downregulate EZH1 expression in MG and stimulate M2-like polarization [164], those enriched in miRNA-3184-3P block the NF-κB axis and also induce M2-like polarization in macrophages [156]**,** which become tolerogenic against the tumor. In addition, when glioblastoma-derived EVs enriched with miRNA-451/miRNA-21 are taken up by MG, these cells experience a downregulation in c-MYC expression and a concomitant upregulation of Arg1 levels, both of which are characteristic features of an M2-like switch [142]. This immunosuppressive TME is further enhanced by the blockade of T cell clonal expansion via CD73, a marker commonly found on the surface of exosomes regardless of their cargo. CD73 was also one of the first discovered MG activation markers [64]. CD73 promotes the production of adenosine, blocking T cell aerobic glycolysis, fostering T cell starvation and, therefore, limiting their entry into the cell cycle [146]. However, astrocytes stimulated by glioblastoma-derived EVs enhance their metabolic fitness state by increasing both glycolytic and mitochondrial energetic routes, reprogramming states that are linked to the neoplastic growth of hypothetically targeted astrocytes [163].

Interestingly, bioengineered EVs show promise as a novel therapeutic approach against malignancies. M1-type MG-derived EVs loaded with ferritin ablate the metabolism of glioblastoma cells by simultaneously blocking lactate efflux and depriving cancer cells of glucose through increased consumption. These events are linked to the production of H_2_O_2_, a switch to an M1-like phenotype, and the induction of apoptosis in glioblastoma cells, suppressing glioma growth in xenograft models in vivo [77].

Tumor-associated foam cells (TAFs) are lipid droplet-loaded macrophages that contribute to a glioblastoma's complex microenvironment [48]. They exhibit pro-tumorigenic characteristics and correlate with poor patient outcomes. Notably, TAF formation can be disrupted by targeting key enzymes involved in lipid droplet formation, such as diacylglycerol O-acyltransferase or long-chain acyl-CoA synthetase [48]. By inhibiting lipid droplet formation, it may be possible to create engineered therapies that selectively disrupt TAF functionality, offering a new avenue for glioblastoma treatment.

## Therapeutic modulation of TAMs in glioma

### Therapeutic interventions alter the function of TAMs, providing novel opportunities

*Section summary* *:* *Therapeutic interventions targeting TAMs in malignant gliomas have been found to reprogram these immune cells, leading to unforeseen consequences. Nevertheless, this phenomenon also unlocks novel therapeutic options, offering strategies for making use of the interplay between TAMs and the glioma TME.*

The behavior of MG and macrophages in high-grade gliomas shows significant differences depending on tumor compartment and progression over time. In areas surrounding blood vessels or zones characterized by necrosis or hypoxia, the cells may display increased mobility, enhanced phagocytic activity, and elevated cytokine release in response to extracellular ATP and purinergic signaling. As the glioma progresses, TAMs can promote angiogenesis, facilitate tumor invasion through the production of matrix metalloproteinases, and suppress immune responses. However, therapeutic interventions such as radiation, chemotherapy, and immunotherapy can reprogram TAMs, sometimes leading to unintended consequences. For instance, radiation therapy can recruit myeloid cells to the tumor and change the functional states of resident MG, leading to priming/training innate immune memory [145]. Likewise, anti-angiogenic therapies can also increase TAM numbers [78]. Recently, time-resolved single-cell transcriptomics (Zman-seq) revealed that monocytes, soon after tumor infiltration, embark on distinct differentiation trajectories upon experimental immunotherapy [58].

Data from preclinical models and human postmortem brain tissues from patients with brain tumors suggest that cancer treatment can profoundly impact the neuroimmune response [1, 132] leading to persistent changes and mediating neurotoxicity. However, further data mapping the dynamic changes of TAMs within the TME after treatment is needed. Importantly, corticosteroids that are routinely administered to patients with glioma to reduce vasogenic edema induce an immunosuppressive program in macrophages [84].

Radiotherapy permeabilizes the BBB and can attract MG and macrophages to glioma [67], where they contribute to treatment resistance by promoting the formation of new blood vessels and enhancing tumor invasiveness through signaling pathways such as SDF-1 (Stromal cell-derived factor-1, known as CXCL12)/CXCR4 and HIF1α [42, 46]. Accordingly, combining radiotherapy with inhibitors that target these pathways may lead to improved treatment outcomes. The efficacy of radiotherapy varies with the radiation modality, e.g., proton therapy, photon therapy, or ultra-high-dose-rate radiotherapy (FLASH–RT) [92], and the level of hypoxia in the tumor core, which can reduce the efficacy of reactive oxygen species generation by radiotherapy.

### Turning immune suppression into immune stimulation is key to unlocking more effective glioma treatments

*Section summary: Targeting microglia and macrophages offers a promising strategy to transform their immune-suppressive roles into immune-stimulatory functions, potentially enhancing glioma treatment efficacy. Although these myeloid cells play a significant role in promoting tumor growth and conferring therapy resistance, targeted interventions—such as manipulating key signaling pathways or exploiting their distinctive receptor expression profiles—make novel therapeutic approaches a reality.*

The role of MG and TAMs in shaping the outcomes of treatments is a new and important aspect that must be considered. These cells facilitate tumor growth and invasion by releasing factors such as TGF-β1 and MMPs like MMP-2 and MMP-9. The presence of these factors enhances the invasiveness of glioma cells and renders them more resistant to therapy. MG and macrophages diminish the effectiveness of chemotherapeutic agents. To counteract these effects, microglial pathways may be targeted using drugs like minocycline [83, 118]. However, a cautionary note is warranted: blocking TAM pathways, such as those initiated by CSF1R signaling [104], can result in the activation of compensatory salvage pathways (e.g., IGF1 signaling [106] or GM-CSF [60]) that ultimately render the tumor resistant to the initial anti-TAM therapy.

In the context of targeted and immunotherapies, MG and macrophages create an environment that limits the success of these treatments by suppressing the immune system. However, in spite of their negative impact on therapies, MG and macrophages also offer novel opportunities for therapeutic intervention. For instance, their expression of specific receptors, such as folate receptor β, can be exploited to deplete or modulate their function. Additionally, therapies that activate MG, such as TLR3 agonists or IL-12, have the potential to enhance anti-tumor immune responses. It has been reported that FLASH radiotherapy (ultra-high-dose-rate irradiation) reprograms macrophage lipid metabolism leading to a reversal of tumor immunosuppression and sensitizing another CNS tumor type, medulloblastoma, to CAR-T cell immunotherapy [89]. Similarly, the use of low-dose radiation has been shown to enhance the efficacy of GD2 TRAC-CART cell therapy in neuroblastoma [131].

### Targeting TAMs in glioblastoma therapy

*Section summary: Targeting TAMs in glioblastoma has proven challenging due to their heterogeneity and plasticity, with many clinical trials showing limited success. However, emerging strategies such as epigenetic reprogramming, SMAC mimetics, and non-steroidal anti-edema therapies offer new potential to modulate TAM functions and enhance treatment efficacy.*

**Fig. 2:**
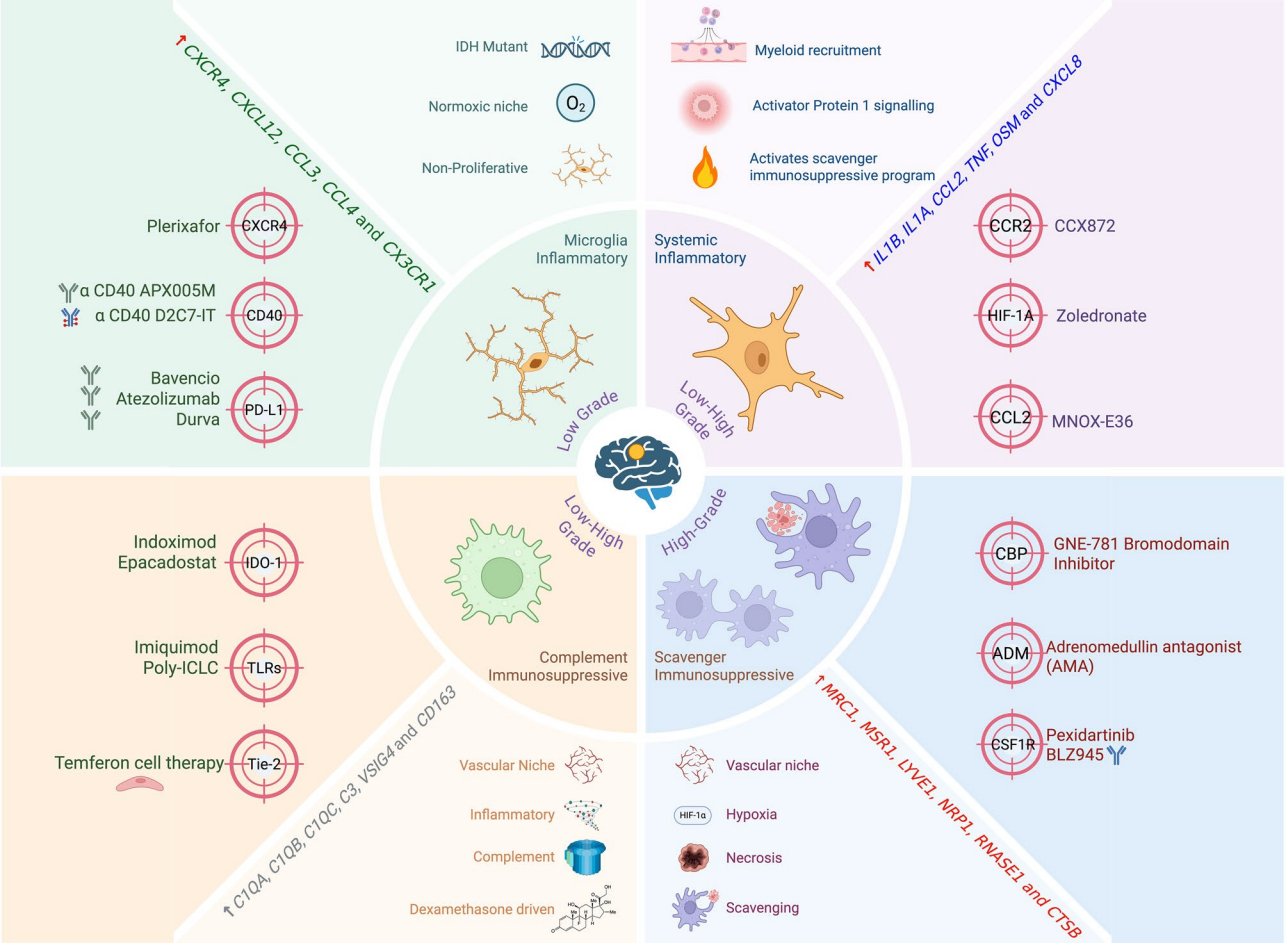
Immune programs across low- to high-grade gliomas and their targetable pathways. Schematic illustrating four major myeloid and glial immune programs [84] that emerge across the spectrum of low- and high-grade gliomas. Each quadrant represents a dominant transcriptional and functional state with characteristic genes, microenvironmental drivers, and available therapeutic strategies. *Microglia-Inflammatory Program (low-grade enriched):* This state is prominent in low-grade gliomas and features resident microglial activation with markers such as *CXCR4, CXCL12, CCL2, CCL3, CCL4, and CX3CR1*. These cells maintain a normoxic, IDH-mutant, non-proliferative niche. Targeted pathways include the CXCR4 axis (plerixafor), CD40 agonists (APX005M, D2C7-IT), and PD-L1 blockade (atezolizumab, durvalumab). *Systemic-Inflammatory Program (low- to high-grade transition)*: This program reflects strong cytokine-driven inflammation influenced by myeloid recruitment and activator protein 1 signaling. Key associated genes include *IL1B, IL1A, CCL2, TNF, OSM, and CXCL8*. These features become more pronounced with increasing grade and correlate with microenvironmental stimulation. Potential interventions include bromodomain inhibition (GNE-781), adrenomedullin antagonists (AMA), and CSF1R inhibitors such as pexidartinib and BLZ945. *Complement-Immunosuppressive Program (observed in both low- and high-grade gliomas)*: This state is defined by complement activation and immunoregulatory signaling, enriched for *C7, C4A, C1QB, C1QC, C3, VSIG4, and CD163*. These cells often arise in niches shaped by vascular and inflammatory cues as well as dexamethasone exposure. Targetable pathways include IDO1 (indoximod, epacadostat), TLR agonists (imiquimod, poly-ICLC), and Tie-2-directed approaches such as temferon cell therapy. *Scavenger-Immunosuppressive Program (predominantly high-grade)*: A high-grade associated program driven by hypoxia, necrosis, and scavenger receptor activity. Marker genes include *MARCO, MSR1, LYVE1, NR4A1, RNASE1, and CTSB*. These features reflect adaptation to metabolic stress and a strong immunosuppressive microenvironment. Targetable mechanisms overlap with those affecting CSF1R signaling and adrenomedullin modulation, as indicated in the therapeutic panel. Created with BioRender. Courtesy A. Anand/B.W. Kristensen

Despite the abundance of TAMs in glioblastoma, attempts to therapeutically reprogram them have so far delivered little clinical benefit. A recent census of the clinicaltrials.gov database identified 54 myeloid-based strategies tested in glioblastoma; however, only three phase-III trials (CheckMate 143, 508, and 548) have been completed, and all three were negative [3]. Early-phase studies of CSF-1R inhibitors illustrate the difficulty: the small-molecule Pexidartinib can cross the BBB and deplete circulating monocytes, yet it failed to produce objective responses in recurrent glioblastoma. Similarly, the CSF1R antibody BLZ945 showed only limited efficacy when combined with PD-1 blockade. Other myeloid-depletion strategies, such as low-dose capecitabine to reduce MDSCs or CXCR4 antagonists like plerixafor to inhibit monocyte recruitment, remain in early clinical testing [3] (Fig. 2).

Preclinical evidence suggests that agonistic CD40 antibodies can reprogram glioma-associated macrophages towards an anti-tumor phenotype [143]. In experimental glioma models, these agents synergized with CSF1R blockade, IL‑6 inhibition and the microtubule‑disrupting agent lisavanbulin (BAL101553) [43], highlighting their potential to overcome immune-checkpoint inhibitor resistance. These findings have informed two ongoing phase‑I trials in glioblastoma: one assessing APX005M (sotigalimab) monotherapy (NCT03389802), and another testing the Fc–engineered anti–CD40 agonist 2141‑V11 in combination with the dual‑specific immunotoxin D2C7‑IT (NCT04547777). Macrophage‑based cell therapies (CAR‑macrophages) [76, 168] and metabolic checkpoint inhibitors (IDO1 blockers) are all being evaluated, but none has yet produced durable survival improvements (NCT02327078; NCT04047706, NCT02764151) [74]. Moreover, blockade of the CD47/SIRPα “don’t eat me” signal with magrolimab was recently halted due to excess mortality (phase III-NCT05079230), underscoring the on‑target toxicities that plague myeloid‑directed therapies.

## Myeloid influence on anticancer therapy

A central reason for these failures is the remarkable heterogeneity and plasticity of TAMs. Single-cell transcriptional deconvolution has revealed that MG and infiltrating monocytes actuate four immunomodulatory programs, microglial inflammatory, systemic inflammatory, scavenger immunosuppressive, and complement immunosuppressive [84] (Fig. 2). These programs are spatially distinct within glioma niches and shaped by the TME; for example, the complement-immunosuppressive program is driven by glucocorticoid-receptor signaling, which can be triggered by endogenous corticosteroids and is markedly upregulated by dexamethasone [84]. Dexamethasone, routinely used to control cerebral edema, simultaneously promotes abnormal myeloid proliferation and expansion of myeloid-derived suppressor cells in the bone marrow, thereby worsening systemic immunosuppression. Moreover, the presence of myeloid cells has been implicated in conferring resistance of malignant cells to chemotherapy in glioblastoma [134] and other cancer types [22, 28, 51], further complicating therapeutic efficacy. High CD204^+^ TAM density was found to correlate with higher tumor grade and was independently associated with poorer survival in glioblastomas treated with temozolomide and irradiation, supporting a pro-tumorigenic role for M2-like macrophages in glioblastoma progression [133]. These findings highlight the need to replace dexamethasone with more immunologically neutral anti-edema strategies such as anti-VEGF therapy [66]. Moreover, to mitigate steroid-associated toxicities, several non-steroidal strategies are under active investigation. Among the most promising are inhibitors of RAGE and S100A9, such as azeliragon and tasquinimod, which have demonstrated the ability to suppress inflammation and preserve blood–brain-barrier integrity [111]. In preclinical surgical brain-injury models, genetic or pharmacologic blockade of RAGE or S100A9 reduced cerebral edema to a degree comparable to dexamethasone, while maintaining the efficacy of concurrent anti-PD-1 immunotherapy [70].

Early-phase clinical trials are now evaluating the translational potential of these approaches. One ongoing Phase I/II study (NCT05773664) is testing peri-operative administration of azeliragon as a targeted RAGE inhibitor to prevent postoperative edema. A separate Phase I dose-de-escalation study is assessing whether combining azeliragon with low-dose dexamethasone can achieve adequate edema control while enabling significant steroid sparing. Additionally, a Phase I trial pairing azeliragon with stereotactic radiosurgery reported that oral RAGE inhibition could safely substitute for corticosteroids, achieving robust clinical responses without dose-limiting toxicities [62].

Moreover, mechanistically informed interventions have begun to emerge. In patient-derived organoids, the selective p300/CBP bromodomain inhibitor GNE‑781 downregulated the scavenger immunosuppressive program and associated AP‑1 transcriptional signatures while restoring a default inflammatory profile [84]. This suggests that epigenetic reprogramming may tilt TAMs away from tumor‑supportive states. Similarly, the CNS‑penetrant SMAC mimetic GDC‑0152 reprogrammed MG and monocyte‑derived macrophages in ex vivo glioblastoma explants and mouse models [12, 130]. The treatment promoted microglial activation, enhanced antigen presentation, reduced anti‑inflammatory macrophages, remodeled vasculature and increased CD8 T‑cell infiltration, collectively converting an immunosuppressive microenvironment into an anti‑tumor one. These proof‑of‑concept studies indicate that targeted perturbation of specific transcriptional programs or apoptotic pathways can overcome intrinsic myeloid plasticity.

In summary, the clinical setbacks of CSF‑1R inhibitors, immune‑checkpoint blockade and other non‑discriminatory strategies underscore the need for next‑generation therapies that integrate the diverse phenotypes and programs of TAMs [162]. Rational combinations that couple epigenetic or apoptotic modulators (such as p300/CBP inhibitors or SMAC mimetics) with agents that block glucocorticoid‑induced immunosuppression, restrain systemic myelopoiesis and enhance lymphocytic infiltration may ultimately produce more durable responses in glioblastoma. Future approaches could use combination therapies of macrophage-based cellular immunotherapies and immunocytokines [75].

## Summary of key points of this statement and implications for our understanding of microglial and macrophage functions in gliomas

- There is cellular, spatial, and temporal heterogeneity of TAMs in gliomas
- MG and monocyte-/bone marrow-derived macrophages can exert tumor-supportive or anti-tumor functions and are highly plastic, which makes them difficult to distinguish and target
- The spatial distribution of MG and macrophages in gliomas plays a role in their functional behavior
- Glioma cells manipulate TAMs to suppress anti-tumor functions and maintain tumor-supportive functions that help the persistence of GSCs
- MG functions converge towards those of classical activated macrophages in gliomas
- MG and macrophages in glioma-affected brains exhibit a spectrum of functional states that are plastic, modulated by the tumor, and differ from the conventional M1/M2 paradigm
- Reciprocal molecular interactions between TAMs and GSCs sustain stemness, drive invasion, and foster therapeutic resistance in glioblastoma
- MG-mediated modulation of neuron-glioma cell interactions is a novel mechanism of tumor promotion
- MG-derived EVs regulate neuronal activity and synaptic plasticity
- Glioblastoma-derived EVs reprogram MG to support tumor progression
- Bioengineered EVs are a novel approach to targeting glioblastoma
- TAMs are key players in tumor angiogenesis and vessel remodeling in gliomas
- Therapeutic interventions alter the function of TAMs, causing unintended consequences but also providing novel opportunities to attack the tumor
- Turning immunosuppression into immunostimulation may be key to unlocking more effective glioma treatments
- Abandon simplistic and/or misleading terms such as “M1/M2” and “neuroinflammation” which stand in the way of research progress (see Textboxes 1 and 2)

**Textbox 1:**
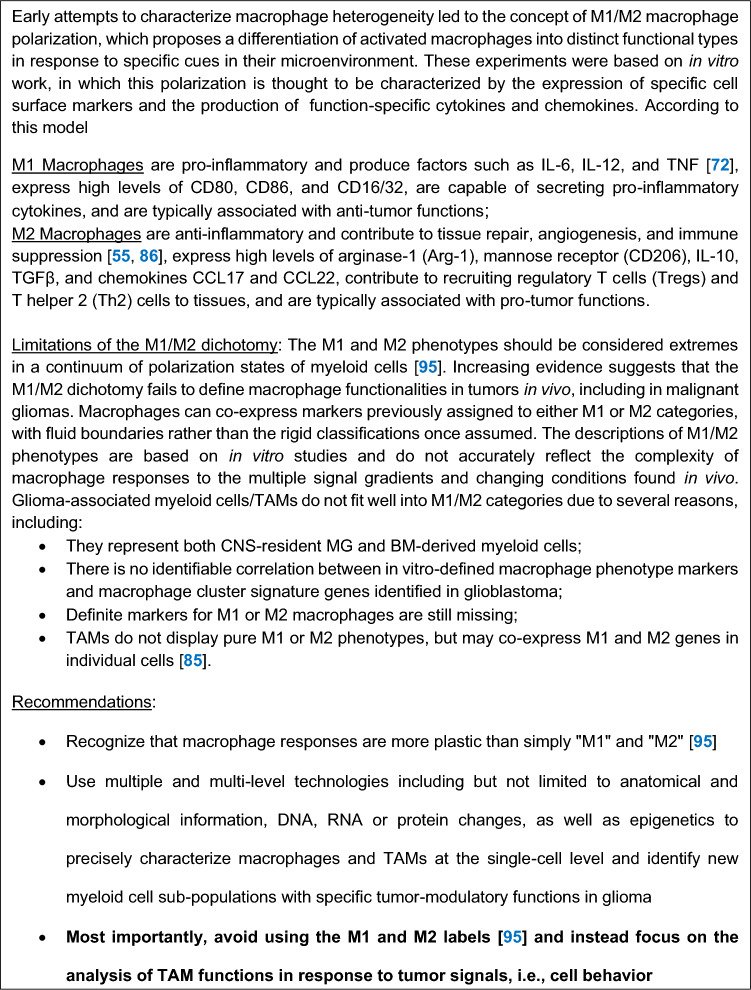
Avoid using M1 and M2 labels

**Textbox 2:**
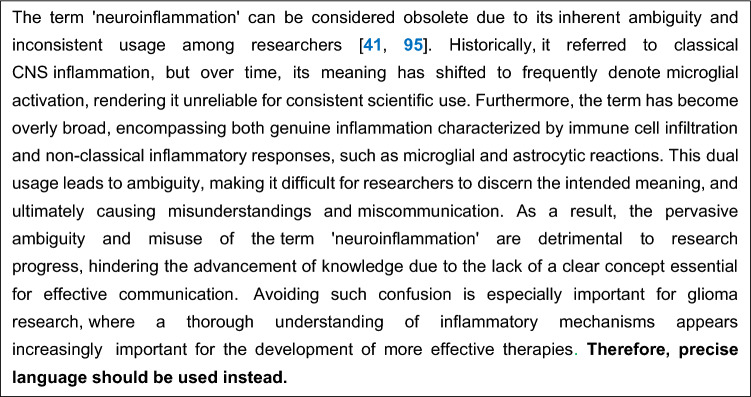
Retiring the term neuroinflammation

## Suggestions for future research directions: TAMs as delivery systems for glioma therapies

Understanding the role of MG and macrophages in the context of tumor treatment is critical. For example, it is important to consider the clear differences between intratumoral MG and TAMs or MDSCs, as the latter are not directly exposed to radiation therapy. This realization may give rise to new treatment approaches. Particularly, the recruitment of monocytes to the tumor following irradiation could potentially be exploited as a Trojan horse strategy. A promising approach involves engineering MG and macrophages to serve as delivery vehicles for nanoparticles, immunocytokines, or other therapeutic agents.

Because access to post‑treatment human brain‑tumor tissue is limited—specimens are obtained at surgical resection before adjuvant therapies are initiated—studying the early effect of treatment directly in patients is challenging. This limitation underscores the need for advanced models that recapitulate the human glioma TME, such as induced pluripotent stem cell‑derived cerebral organoids incorporating MG and vascular cells [108, 117].

There is a pressing need for research on TAMs in therapeutic targeting and timing, given the absence of specific treatments and the limitations of non-specific approaches that target entire signaling axes, such as CSF1/CSF1R or CD47/SIRPα, which can lead to treatment resistance or off-target results [18, 24, 104, 106]. In addition, the timing of the treatment is crucial in modulating tumor control and subsequent toxicity. SMAC mimetics act on the TAMs compartment, promoting anti-tumoral response and reducing immunosuppression [130, 154]. Recently, engineered macrophages endowed with adaptative polarization have been efficiently used to deliver a radiosensitizer to glioblastoma [37].

## Important note

While this manuscript has elements of a review article, it was not designed as such but rather as a consensus statement. For this purpose, the group first agreed on a list of key topics that were deemed particularly relevant. These topics were discussed during an online meeting that had high attendance. Subsequently, the initiating group drafted a manuscript with limited references. The text was then reviewed and edited online by all participants, retaining only those points that were found to be both important and well-supported in the scientific literature. Some of these points received additional attention and were elaborated upon further. When disagreements arose, formal votes were held—both during the conference and in subsequent email discussions. Therefore, this consensus statement does not aim to cover every aspect of the microglia/TAM literature and glioma but instead focuses on those points that are widely agreed upon.

## Data Availability

No datasets were generated or analysed during the current study.

## Abbreviations

2-HG: 2-Hydroxyglutarate
BBB: Blood–brain barrier
CCL2: C–C motif chemokine ligand 2
CSF1: Colony-stimulating factor 1
CSF1R: Colony-stimulating factor 1 receptor
ECM: Extracellular matrix
EVs: Extracellular vesicles
GSCs: Glioma stem cells
IDH: Isocitrate dehydrogenase
MDMs: Monocyte-derived macrophages
MDSCs: Myeloid-derived suppressor cells
MG: Microglia
MMPs: Matrix metalloproteinases
SIRPα: Signal-regulatory protein α1
TAMs: Tumor-associated macrophages/microglia
TGF-β: Transforming growth factor β
TME: Tumor microenvironment
